# Encoding and Multiplexing of 2D Images with Orbital Angular Momentum Beams and the Use for Multiview Color Displays

**DOI:** 10.34133/2019/9564593

**Published:** 2019-02-12

**Authors:** J. Chu, D. Chu, Q. Smithwick

**Affiliations:** ^1^Centre for Photonics Devices and Sensors, University of Cambridge, 9 JJ Thomson Avenue, Cambridge CB3 0FA, UK; ^2^Disney Research, Glendale, California 91201-5020, USA

## Abstract

The orthogonal nature of different orbital angular momentum modes enables information transmission in optical communications with increased bandwidth through mode division multiplexing. So far the related works have been focused on using orbital angular momentum modes to* encode/decode and multiplex point-based on-axis signals* for maximum data channel numbers and capacity. Whether orbital angular momentum modes can be utilized to encode/decode* off-axis signals* for multiplexing in two-dimensional space is of significant importance both fundamentally and practically for its enormous potential in increasing the channel information capacity. In this work, a direct use of orbital angular momentum modes to* encode/decode and multiplex two-dimensional images* is realized in a scalable multiview display architecture, which can be utilized for viewing three-dimensional images from different angles. The effect of off-axis encoding/decoding and the resultant crosstalk between multiplexed different two-dimensional views are studied. Based on which, a color display of good image quality with four independent views is demonstrated. The resolution of the decoded images is analyzed and the limitation of this approach discussed. Moreover, a spatially multiplexed data communication scheme is also proposed with such a two-dimensional encoding/decoding approach to significantly enhance the data transmission capacity in free space for future data communication needs.

## 1. Introduction

Different techniques have been investigated in order to display a three-dimensional (3D) image, through direct image reconstruction using light fields [1] and holographic methods [2, 3]. Alternatively, a 3D image can be mimicked by displaying appropriate discrete two-dimensional (2D) images. Layer-based methods [4] project multiple 2D images at depths of a 3D image, while stereoscopic displays [5] and multiview displays [6] project discrete 2D images viewed at different angles. Stereoscopic 3D displays benefit from the convergence cue of human vision and deliver two images to the two eyes. Among them, those based on polarization-division multiplexing [7] are limited fundamentally to two views because the information is encoded with one of the two orthogonal polarization states. For those color-interlaced (anaglyph) displays [8], the images have to be encoded for the left- and right-eyes separately with two different near-complementary colors. Time-multiplexed stereoscopic displays utilize the visual persistence of vision [9] and display two images sequentially for two eyes, respectively. However, the number of full-resolution views that can be projected at the same time is limited due to the physical properties of polarization and anaglyph. Similarly, multiview displays which use time-sequential [10] or spatial multiplexing [11, 12] approaches for separation of different images suffer from the trade-off between image resolution and number of views projected simultaneously. All these approaches intend to deliver sufficient amount of information, which is also of interest in a more general context.

As an additional degree of freedom, orbital angular momentum (OAM) of light in theory allows encoding/decoding and multiplexing/demultiplexing virtually unlimited number of channels in communications [13–16]. Transmitting, rendering, and delivering information may also benefit from the theoretically unlimited number of orthogonal OAM modes [17] as communications do. Such information can be 2D images for multiview displays. It was recognized that laboratory light beams having spiral phase with *l* intertwined helices in unit wavelength possess an OAM of *lħ* per photon, where *l* is the OAM mode index [18]. The phase dislocation and on-axis phase singularities of OAM beams open a dark region on the beam axis with zero real and imaginary parts [19, 20]. Because of the orthogonality at the beam axis, OAM beams have been used to encode and decode point-based data with either the on-axis amplitude [13–16] or the mode of an OAM beam [21]. This approach has been extended to encode and decode 2D images by using a pixelated array of OAM modes [21], where a 2D was deciphered into an array of pixels and each pixel was treated as an on-axis point to be encoded and decoded by a spatially separated OAM mode. By creating an array of OAM beams, encoding and decoding of a 2D image rendered as 9x9 pixels with all the pixels deciphered simultaneously were demonstrated [22, 23]. However, the information encoded here with OAM is fundamentally on-axis and, therefore, point-based and it will be extremely difficult to scale up this approach for the applications of high resolution 2D images.

In order to develop a practical approach to encode/decode 2D images using OAM modes, coding of not only the on-axis point but also all the off-axis points should be considered. Recently, we investigated mathematically the general situation of encoding and decoding 2D information with OAM beams [24, 25]. Large dark regions introduced by large OAM modes suggest that 2D images can be decoded and spatially separated from vortices [24]. Furthermore, we showed that an off-axis point can be encoded/decoded by an OAM spectrum instead of a single mode [25], similar to the case of misaligned OAM beams [26, 27]. Consequently, it is possible to use some carefully chosen OAM modes to encode/decode as well as multiplex/demultiplex 2D view images comprising both the on-axis point and off-axis points.

Here, we propose the use of OAM modes to* encode/decode and multiplex 2D images* and demonstrate it in a novel multiview display architecture which is capable of delivering high quality color images for viewing three-dimensional (3D) images from different angles. Experimental setup is constructed to demonstrate its performance for four independent views, with the largest coded OAM mode of 12 and an effective coding area of 800 *μ*m. This breaks the limit of conventional two-view autostereoscopic displays using polarized lights. Our experiment confirms the possibility of increasing information coded with OAM modes from point-based to 2D with predictable and controllable crosstalk, which is in good agreement with our theoretical analysis. The use of OAM modes to encode/decode and multiplex 2D information in free space optical data communication is also proposed to significantly enhance the data transmission rates for future demands.

## 2. Results

### 2.1. Encoding 2D Images with OAM Spectra

**Figure 1:**
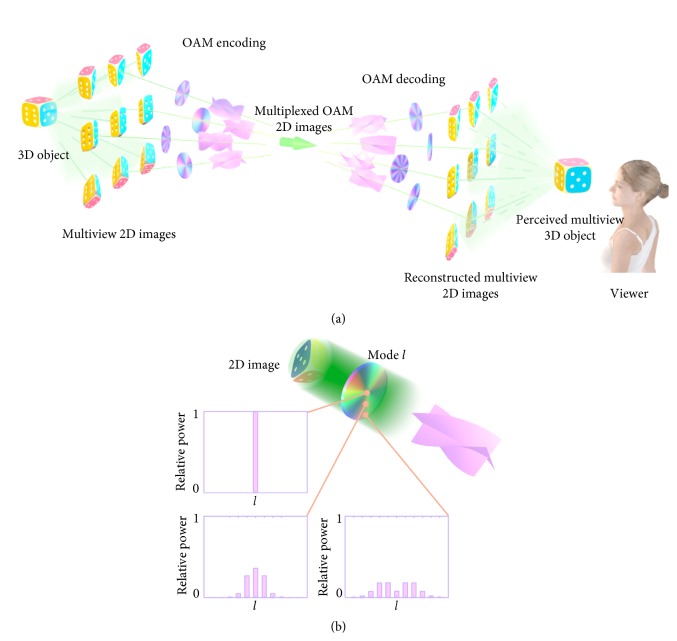
Encoding/decoding images with different OAM modes for multiview displays. (a) Schematic illustration of multiview display using OAM modes for a 3D object. (b) Schematic of the resultant OAM spectra as an effect of encoding a 2D image.

Figure 1(a) illustrates schematically the proposed multiview architecture based on coding/decoding multiple 2D view images with OAM beams. Information of 2D images viewed from different angles of a 3D image is carried by the multiplexed view-coded OAM beam, where each image is modulated by a helical phase plate which has a unique number of intertwined helices and handedness. A 2D image consists of the on-axis point and multiple off-axis points. In a view, the on-axis point of an image is coded with the OAM mode of the light beam as that in conventional cases [13, 16, 21–23]. OAM of light is defined with respect to an arbitrary axis [27]. When defined with respect to an off-axis point, an on-axis OAM beam can be regarded as superposition of an infinite set of off-axis OAM modes with different modes [25]. Encoding/decoding an off-axis point is encoding/decoding an OAM spectrum comprising a set of OAM modes which are symmetrical with respect to the on-axis OAM mode (Figure 1(b)).

Unlike perfectly aligned OAM modes for on-axis coding, encoding/decoding off-axis points for multiplexing can introduce crosstalk. On the condition that the OAM spectra encoded with any two different images are not sufficiently separated, the overlapped components in the OAM spectra result in crosstalk between the two images. Because most of the energy in an OAM spectrum is focused close to the central mode, separation of OAM spectra and effective coding area to avoid crosstalk can be demonstrated by separation of the central modes. Here intensities of the central component in an OAM spectrum are experimentally resolved and measured by encoding/decoding a ring (derivations in Materials and Methods).

**Figure 2:**
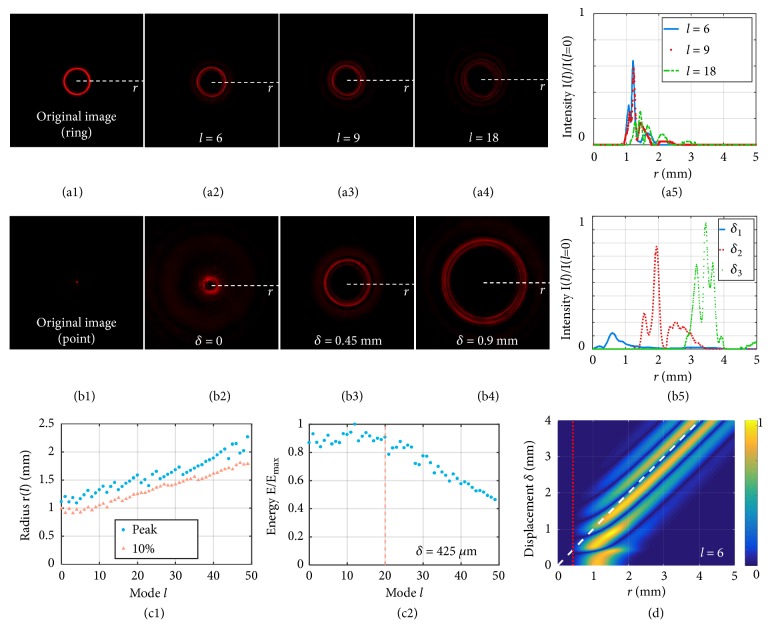
Validation of 2D encoding theory. (a) Measured intensity of the central component in an OAM spectrum by coding an on-axis light feature of 50 *μ*m width with a constant off-axis displacement* δ* (i.e., ring radius) of 0.425 mm using different on-axis OAM modes: (a1)* l*=0 (original image), (a2)* l*=6, (a3)* l*=9, (a4)* l*=18, and (a5) radial intensities of Figures 2(a2)–2(a4). (b) Measured intensity of the central component by coding a light feature of 100 *μ*m width with different constant off-axis displacements from the axis of a given OAM mode: (b1) original image of the point at* δ*_1_=0 without OAM coding, (b2) a point at* δ*_1_=0 coded with mode* l*=6, (b3) a ring with* δ*_2_=0.45 mm coded with mode* l*=6, (b4) a ring with* δ*_3_=0.9 mm coded with mode* l*=6, (b5) radial intensities of Figures 2(b2)–2(b4). (c) Measured light intensity of the central component as a functional of radial distance* r* from the on-axis point for different modes* l*: (c1) radius defined at 10% and 100% of the peak energy and (c2) the relative energy to the input maximum. (d) Calculated radial intensity of the central component for different off-axis displacements.

Firstly, a light feature of 50*μ*m-wide ring with a constant off-axis displacement* δ* from the light axis (ring inner size *δ*_1_ = 400*μm*, and outer size *δ*_2_ = 450*μm*) was experimentally coded with different modes* l*, and the change of intensity of the main component in an OAM spectrum versus different on-axis coded modes* l* was studied (Figure 2(a)). The intensity profiles of the imaging plane when *l* = 6,9, 18 are shown in Figures 2(a2)–2(a4), and their cross-sections are shown in Figure 2(a5). The intensities create a circular dark region in center, which can be regarded as useful coding area to project an image. Radius of the dark region increases with increased mode* l*, and maximum intensity decreases at the same time. Experimental results of radius of the dark region defined at 10% of the maximum intensity, and radius at maximum intensities versus different modes* l* are plotted in Figure 2(c1). A larger mode* l*, which suggests a larger mode separation between any two of the multiple views in our demonstration, is advantageous for reducing crosstalk in two ways: (1) enlarging effective coding area and (2) spreading the crosstalk energy over a larger area so that the amplitude of crosstalk is low enough to make useful image stand out. As the mode increases, the radius of peak intensity can correspond to a different peak in a typical radial intensity curve as shown in Figure 2(b5). Resultantly, radius of the peak intensity increases in steps. Figure 2(c2) confirms energy integrated over the imaging plane is conserved for* l* ≤ 20. Angular resolution of the spiral phase profile imposed by the liquid crystal on silicon (LCOS) spatial light modulator (SLM) is limited by the large pixel size, and generating OAM beams with larger mode indices is more problematic. In an OAM spectrum, change of weights of the other components versus different modes* l* mimics that of the main component. Taking the less dominant components into consideration, the theoretical effective coding area for a range of different modes* l* can be found in [25]. The fact that the radius of the central component in a spectrum increases with the OAM mode suggests that the effective encoding area increases at the same time.

Secondly, light features with different constant off-axis displacements (i.e., different ring sizes) were coded with the same mode “*l*=6”, and the position and intensity of crosstalk raised in the imaging plane were studied (Figure 2(b)). The width of the rings was 100 *μ*m, and ring sizes of them were “*δ*=0, 450, and 900 *μ*m”, respectively. In the imaging plane, intensities profiles are shown in Figures 2(b2)–2(b4) and the radial intensities are plotted in Figure 2(b5). For completeness, Figure 2(d) shows the calculated cross-sections for the rings with other off-axis displacements* δ*, where the rows agree with measured radial intensities. The red dotted line shows a minimum inner radius of the crosstalk term defined at 10% of the maximum intensity. The white dashed line shows the radii themselves. For encoding/decoding an image having multiple points with a range of different displacements, an effective coding area within which any points in the reconstructed image are placed can be suggested by the triangle formed with the white dashed line and the red dotted line. Encoding/decoding points with displacements smaller than the effective coding area are advantageous to keep low crosstalk. When |*l*| increases, vertex of the triangle would move toward the top right and the effective coding area would increase. In the effective coding area, peaks of the crosstalk term occur where there is discontinuity in the encoded image, and those peaks were used for the edge detection in microscopy [28, 29].

### 2.2. Decoding Multiplexed OAM 2D Images

**Figure 3:**
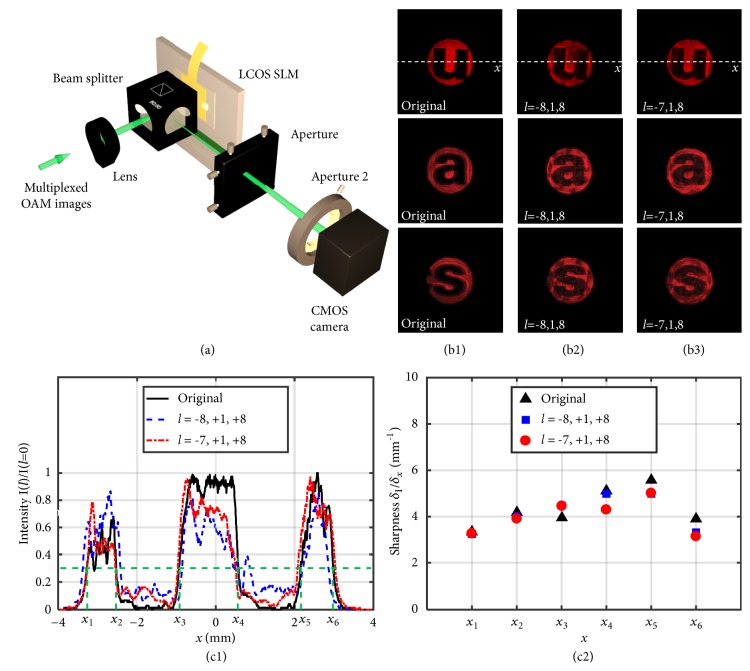
Images decoded from a multiplexed OAM beam and their qualities. (a) Experimental setup. (b) Experimental intensity profiles of images: (b1) diffracted by blazed grating, (b2) reconstructed images encoded and decoded by modes* l*=*∓*8, ±1, ±8, (b3) reconstructed images encoded and decoded by modes* l*=*∓*7, ±1, ±8. (c) Image quality evaluated by normalized intensity variation of the character “u” in Figure 3(b): (c1) horizontal intensity variation and (c2) sharpness calculated from Figure 3(c1).

Experimentally we encoded/decoded images imprinted on chrome masks with helical phase* lφ* imposed by spiral phase plates (SPPs) [17, 30, 31] and LCOS spatial light modulator SLM [17, 32, 33], where* l* is the OAM mode index annotating the handedness and number of helices of the phase front, and *φ* is the angular coordinate in a cylindrical coordinate system. Blocked letters “u”, “a”, and “s” with transparent background were encoded with groups of encoding OAM modes “*l*_*en*_=-8, 1, 8” and “*l*_*en*_=-7, 1, 8”, respectively. The OAM beams containing information of the encoded images were multiplexed and decoded using the experimental setup shown in Figure 3(a). A blazed grating or a spiral phase pattern is loaded on the LCOS. One of the three encoded view images was extracted at a time by the use of a programmable LCOS SLM, which displayed a spiral grating consisting a blazed diffraction grating [34] with a forked dislocation and imposed spiral phase onto the diffraction order [13, 17].

When the decoding OAM mode index* l*_*de*_ matched one of the encoding OAM mode indices* l*_*en*_ so that “*l*_*en*_ +* l*_*de*_ =0”, the interrelated view image was extracted. Encoded with modes “*l*_*en*_=-8, +1, and +8”, the extracted images are shown as follows: Figure 3(b2) shows the three extracted images decoded at the first diffraction order when the OAM mode index of the spiral grating was “*l*_*de*_=+8, -1, and -8”, respectively, and Figure 3(b3) shows the extracted images encoded/decoded with modes *∓*7, ±1, and ±8. For comparison, images diffracted by a blazed grating (without encoding/decoding) are present in Figure 3(b1). In general the quality of a decoded image depends on the OAM mode range used in the encoding and decoding process. Its off-axis points can be affected significantly by the overlapping components in the OAM spectrum from images of other channels which are encoded with adjacent OAM modes. Using an OAM mode further away from the other chosen OAM modes can help to improve the quality of the decoded image and minimize its deviation from its original image. Therefore, it is important to balance mode separation for different images for optimized image qualities.

To analyze image quality, intensity at the horizontal cross-sections of the decoded characters “u” are plotted and compared in Figure 3(c1), and their sharpness calculated from change of intensity in the* x* direction was shown in Figure 3(c2). The outer edges at* x*_1_ and* x*_2_ were cut by a circular aperture and less sharp compared with the other edges which were more related to edges on the image masks. The extracted images encoded/decoded with modes either “*l*=*∓*8, ±1, ±8” or “*l*=*∓*7, ±1, ±8” kept sharp edges compared with the projected image without encoding/decoding. However, energies of the other views went to a circular pattern surrounding the extracted image, and the bright part had lower intensity after normalization. Crosstalk introduced by the other views made the dark part brighter. Image contrast of “*l*=*∓*8, ±1, ±8” is better than that of “*l*=*∓*7, ±1, ±8”, which is consistent with the fact that the larger the mode separation, the smaller the crosstalk noise. Increasing the mode difference among views and improving the alignment may better separate the encoding and decoding OAM spectra, decreasing the imperfectness.

**Figure 4:**
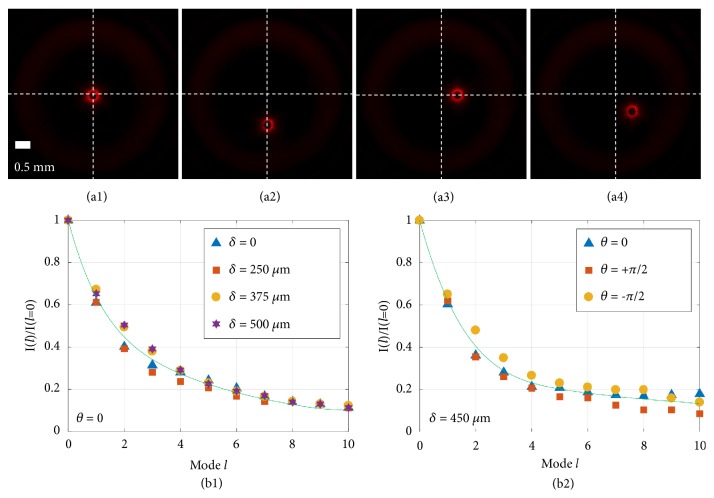
Verification of the integrity of a light beam encoded by different OAM modes on a 2D plane. (a) Shift-invariance demonstrated by intensity profiles recorded at the imaging plane. (b) Shift-invariance validated for different modes: (b1) point encoded at the same polar angle *θ* but a different displacement *δ* and (b2) the same displacement *δ* but a different polar angle *θ*.

The experimental setup decodes off-axis points with on-axis OAM spectra via convolving any point in the encoded image with an off-axis OAM beam (derivations in Materials and Methods), where the term “off-axis OAM” means a nonzero displacement between the OAM's own cylindrical symmetry axis and the experimental system axis. With reference to the experimental system axis, an off-axis OAM beam can be regarded as an on-axis OAM spectrum which consists of a number of different on-axis OAM modes [25]. A small circular region displaced at different positions was used to represent a point and coded with OAM modes.  Figure 4(a) shows typical intensity profiles when an off-axis point is encoded with an OAM mode. Having encoded a 50-*μ*m point at different off-axis positions with the same on-axis OAM mode, the intensity distribution remains the same but its position is shifted depending only on the location of the encoded point.  Figure 4(b) summarizes normalized intensities of the intensity profiles for a range of different modes* l* when off-axis points with different lateral positions and polar angles are encoded. In Figure 4(b1), a 50-*μ*m point was encoded at different lateral displacement, and in Figure 4(b2), a 100-*μ*m point was encoded at different polar angle. The fact that curves describing different off-axis points coincide suggests the shift-invariance property remains for different modes* l*. The process of the convolution employed is therefore indeed shift-invariant.

### 2.3. OAM Multiview Color Display

Among the technologies to pursue projection of realistic 3D images [2, 4, 35, 36], autostereoscopic displays give 3D sensation without the need for viewing glasses [37]. When the number of view images is sufficient, multiview autostereoscopic displays mimic the parallax generated by a realistic 3D object [38, 39]. Supported by the theoretical unbounded state space of OAM, it is possible to encode information of many 2D images and construct an ideal dense multiview display.

**Figure 5:**
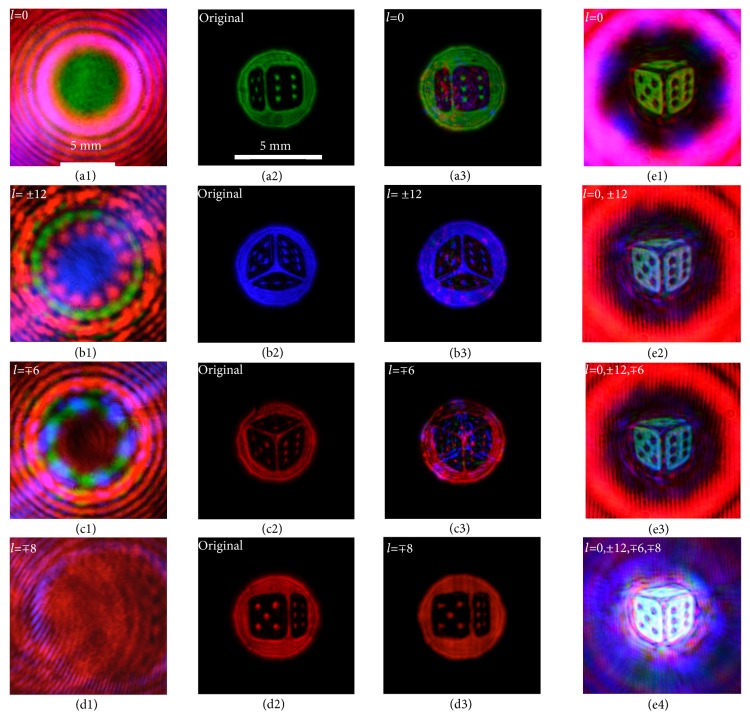
Multiview color display. (a) Right view at 532 nm: (a1) multiplexed OAM beam, (a2) original image without encoding/decoding, (a3) decoded image. (b) Bottom view at 450 nm. (c) Top view at 635 nm. (d) Left view at 632.8 nm. (e) The same image encoded/decoded with different wavelengths but with the decoded images overlaid: (e1) 532 nm only, (e2) multiplexing of 532 nm and 450 nm, (e3) multiplexing of 532 nm, 450 nm, and 635 nm, and (e4) multiplexing of 532 nm, 450 nm, 635 nm, and 632.8 nm.

To demonstrate the concept, we projected a dice consisting of four view images which were decoded simultaneously (Figure 5). The multiplexed view-encoded OAM beam contained information of the left, right, top, and bottom views of a dice which were encoded with OAM modes “*l*_*en*_=-8, 0, +6, and +12”, respectively. Each of the encoded view was decoded from a replication of the multiplexed view-encoded OAM beam and reconstructed. Multiview of a 3D dice could be projected when the decoded views were directed toward the target position from the designed angles, where the horizontal angles were ±20°, and the vertical angles were ±30°. The view images were calculated and imprinted on chrome masks, with the size of 800 *μ*m, and size of the reconstructed view images was 4 mm. Crosstalk was introduced mainly because of adjacent OAM modes used to encode/decode different views. For example, in Figure 5(c3) where the red image decoded by the mode “*l*=-6”, the major crosstalk came from the adjacent OAM mode channel where a blue image was encoded with “*l*=+12”. This can be improved by encoding/decoding some of the images with OAM modes having larger topological charges and increasing mode separation. Recent advance in laser direct nanostructuring of silica glass demonstrates optical elements generating OAM modes of up to “*l*=100” [40], which can potentially allow us to have larger mode separation and reduced crosstalk practically.

Though phase plates are wavelength specific [17], color images can still be encoded and decoded. Figure 5(e) presents a white view image reconstructed by the combination of four wavelengths coding the same view image, where a 632.8 nm image was coded with modes “*l*=*∓*8”, a 532 nm image was coded with the mode “*l*=0”, a 635 nm image was coded with modes “*l*=±6”, and a 450 nm image was encoded with modes “*l*=±12”. The first red channel of 635 nm has a lower intensity than the second read channel of 632.8 nm in order to reduce the crosstalk and achieve the final white image; therefore Figures 5(e2) and 5(e3) appear similar.

### 2.4. Spatially Multiplexed OAM Modes

**Figure 6:**
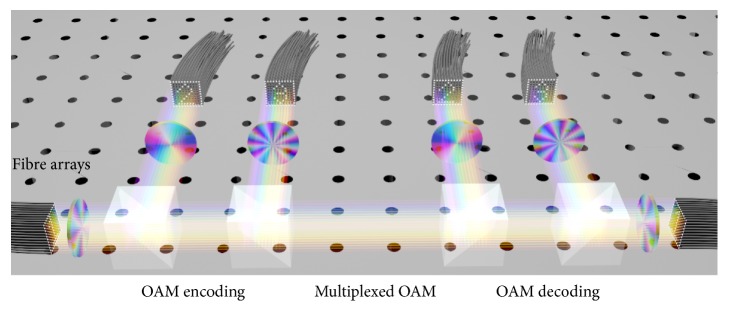
Schematic of encoding, multiplexing, and decoding spatially separated data channels of individual fibre arrays with individual single OAM modes.

The 2D image-based OAM encoding/decoding method described above can benefit a wide range of applications. Here we propose its use in spatially multiplexed data channels in free space to significantly further expand the data transmission capacity. A schematic drawing of such an approach is shown in Figure 6. By carefully positioning the fibre array in 2D and choosing the appropriate OAM modes, independent communication between different 2D fibre arrays with minimum crosstalk can be realized. The use of a single OAM phase plate for each 2D fibre array instead of using it for each fibre in both the sending and receiving ends will be able to reduce the complexity and costs enormously and make this scheme an efficient and viable approach in high bandwidth data communication in free space.

## 3. Discussion

The orthogonal nature of OAM beams possessing different OAM mode indices makes them ideal to support additional degrees of freedom in encoding and decoding information. The Fourier relationship between OAM and the angular position [41, 42] expands an off-axis OAM beam into an OAM spectrum having weighted OAM modes, which suggests an off-axis point can be encoded/decoded with an OAM spectrum. Having proposed to extend conventional point-based on-axis encoding/decoding to 2D based off-axis encoding/decoding, we further suggest that 2D information being encoded/decoded with a single OAM mode for multiplexing can be multiple levels rather than just a single bit as black/white. We demonstrate the idea by encoding different OAM modes with different 2D images. Central components of OAM spectra are measured and analyzed, showing that crosstalk between any two different views can be reduced using sufficiently separated OAM modes or with a reduced image size. Using carefully selected OAM modes, three 2D images were encoded, multiplexed, and decoded with their image quality unchanged. We then demonstrate the use of the proposed multiview color display architecture with four static images and four wavelengths, to illustrate the reconstruction of views of a 3D object at four different angles, which breaks the two-view limit of the polarization multiplexing scheme. Such a multiview static display can be extended easily to a multiview video display by simply replacing the static images in use with video images. Furthermore, it is also possible to scale up the size of the images with larger mode separation and scale up the number of views using more OAM modes. A limitation of the approach lies in that generating higher OAM modes is practically refined by the pixel size of the LCOS SLMs or the accuracy of the SPPs and there is trade-off between image size and number of images for a given set of OAM modes. Mode number as high as ±100 has been realized in silica glass [40]. To further overcome such a limitation in encoding and decoding images, it may be possible to generate even higher OAM modes using metasurfaces [43]. Finally, the use of OAM modes to encode/decode 2D information and multiplex them spatially can be extended to a broader research applications, such as free space optical data communications, where the capacity of data transmission can be further enhanced enormously to meet the future demand of ever growing data transmission rates.

To optimize the mode selection for multiview color display with more views and less parallax within a limited range of OAM modes, the main concern in this case is the crosstalk among different images. This is related to the image size and mode separation: larger images and smaller mode separation would have more crosstalk. For minimum crosstalk on the image quality, consideration of the trade-off between the image size and mode separation (or number of views if absolutely necessary) is needed. Figure 2(c) illustrates the relationship between image size and mode separation. Suppose that we select the OAM modes from a base set of {*l*_1_, *l*_2_, *l*_3_,…} for different views and the separation between any two modes is *l*_i-j_. From Figure 2(c) and related previous results [25], the size of image that can be encoded/decoded is a function of the mode separation: *r*_i-j_*∝l*_i-j_. Based on this set of *l*_i-j_, the resultant number of images for a designed image size is N *∝* 1/r_design_, where N is the number of *r*_i-j_ > r_design_. The appropriate OAM modes from {*l*_1_, *l*_2_, *l*_3_,…} are such that *r*_i-j_ > r_design_.

It is worth mentioning that the absolute amplitude or intensity of encoded light beam does change during the propagation. The information it carries in this case depends on the image contrast, i.e., the spatial distribution of the light intensity, rather than the absolute amplitude itself. The overall change of the amplitude of a single OAM beam transmitted over a long distance will have little effect on the amount of the information it carries. In order to prevent information deterioration, any optical components which may eventually affect the output spatial distribution of light intensity are avoided in the experimental setup.

## 4. Materials and Methods

### 4.1. Experimental Design

A uniform light beam was firstly modulated spatially by a 2D amplitude image and then encoded in an OAM mode by passing a SPP of a certain order. Different OAM encoded beams were then multiplexed spatially before they were split to different directions with each decoded with a corresponding complementary SPP for image reconstruction, as illustrated in Figure 1(a). Three relay lenses were used in the light path. Sizes of encoded images were selected based on size of dark regions of OAM beams generated by the ordered SPPs. Size of the dark region increases with OAM mode |*l*| and confines maximum size of images that can be encoded/decoded for multiplexing (Figure 2). Intensity profiles of light beams were recorded by a CMOS camera with 4928x3264 pixels and 23.6x15.6 mm at the imaging plane. The Figures displayed in this study were cropped for the purpose of demonstration. Experiments for Figures 2 and 4 were repeated three times and the other experiments were repeated more times. The results of the experiments were highly repeatable.

*Light Sources.* A 632.8 nm linearly polarized HeNe laser was used in Figures 2–4. The HeNe laser and 532 nm, 635 nm, and 450 nm laser diodes were used in Figure 5.

*Generation of OAM Beams.* We used SPPs in combination with a blazed grating displayed on a LCOS SLM to impose spiral phase and generate OAM beams. When Figure 5(d3) was extracted, a 0~2.1*π* blazed grating was used to reduce diffraction efficiency of blue/green light so that crosstalk introduced by the blue/green images was minimized.

*Image Formation.* The encoded images and testing targets were chrome masks imprinted on glass, with a pixel size of 2 *μ*m. They were relayed using 4*f* system and then projected using an imaging lens. At the imaging plane, we denote the function of the projected image as* t*_*A*_.

*Performing Convolution of the Encoded Image with the Crosstalk Function.* In Figure 3(a), suppose the distance from the imaging lens to the plane, where the LCOS is placed, is* d*_1_; the distance from the LCOS plane to the imaging plane is* d*_2_; and the focal length of the imaging lens is* f*. The Cartesian coordinate system of the LCOS plane is (*ξ*,*η*), and azimuthal coordinate of the polar coordinate system of that plane is* ϕ*. The Cartesian coordinate system of the imaging plane is (*x*,*y*), and the polar coordinate system of the imaging plane is (*ρ*,*φ*). Using the Fresnel diffraction [28], the complex field of the imaging plane* U*_*d*2,*l*_(*x*,*y*) can be written as a function of the complex field just after the LCOS for plane* U*_*d*1,*l*_(*ξ*,*η*)exp(*ilϕ*) in that

(1) Ud2,lx,y=Ud1ξ,ηexp⁡ik/2d2x2+y2iλd2·exp⁡ikd2×∫∫−∞∞Ud1ξ,ηexp⁡ilϕ·exp⁡ik2d2ξ2+η2·exp⁡−i2πλd2xξ+yηdξdη

where* λ* is the wavelength,* k* is the wave number, and* l* is the azimuthal mode (i.e., the OAM mode) of the overall spiral phase imposed by the LCOS and SPP/SPPs. When the image is encoded with mode* l*_1_ and decoded with mode* l*_2_, the overall spiral phase imposed is “*l*=*l*_1_+* l*_2_”. When “*l*=0”, no overall spiral phase is imposed, and the encoded image is projected, yielding

(2) Ud2,l=0x,y=Ud1ξ,ηexp⁡ik/2d2x2+y2iλd2·exp⁡ikd2×∫∫−∞∞Ud1ξ,η·exp⁡ik2d2ξ2+η2·exp⁡−i2πλd2xξ+yηdξdη=tAx,y.

Because of the shift-invariant property of the Fourier transform and the convolution theorem, the complex field of the imaging plane can be written as the convolution of the encoded image with the crosstalk function so that

(3) Ud2,lx,y=tAx,y∗∫∫−∞∞exp⁡ilϕexp⁡−i2πλd2xξ+yηdξdη.

We define ∫∫_−*∞*_^*∞*^exp⁡(*ilϕ*)exp⁡[−*i*(2*π*/*λd*_2_)(*xξ* + *yη*)]*dξdη* as the crosstalk function of the system, which has been studied in our previous work [25]. Here we use LG beams *E*_*l*_(*x*, *y*) with OAM mode index *l* and beam waist *w*_0_ which affects size of the dark area to model the crosstalk function. Figure 6(b) shows the crosstalk function is shift-invariant.

*Crosstalk between Two Views Is Dependent on the Mode Separation.* Suppose the first view is encoded with mode* l*_*1*_ and decoded with mode -* l*_*1*_. The second view is encoded with mode* l*_*2*_. In the experimental setup, the view-encoded OAM beams are multiplexed after encoding and sent to decoding. In the first decoded view, the crosstalk introduced by the second view is equivalent to the second encoded view modulated by a total mode index “*l* =* l*_*2*_ –* l*_*1*_”.

*Encoding/Decoding an Off-Axis Point Is Encoding/Decoding an On-Axis OAM Spectrum.* As indicated by (3), when* t*_*A*_(*x*,*y*) is an off-axis point, the complex field at the imaging plane is an off-axis crosstalk function, which we model by the use of LG beams here. Suppose position of the off-axis point at the coordinate system (*x*,*y*) is “*x*=*x*_0_”, “*y*=*y*_0_”, and its position at the coordinate system (*ρ*,*φ*) is “*ρ*=*δ*”, “*φ*=*θ*”; then the complex field is* E*_*l*_(*x*',*y*') where “*x*'=*x*-*x*_0_” and “*y*'=*y*-*y*_0_”. Written in the corresponding polar coordinate system (*ρ'*,*φ'*), the off-axis LG beams* E*_*l*_(*ρ'*,*φ'*) can be expanded back to the original coordinate system (*ρ*,*φ*) in that

(4) $$\begin{array}{c} E_{l}\left(\;\rho^{\prime },\varphi^{\prime }\;\right)=\overset{\infty }{\underset{m=-\infty }{\sum }}B_{sgn\left(l\right)m}\left(\;\rho ,\varphi \;\right)E_{sgn\left(l\right)m}\left(\;\rho ,\varphi \;\right) \end{array}$$

where

(5) Bm=l!m!2w0l−mρ−mexp⁡−δ2w02·∑0lClnρn−δl−nIsgnlm−n2ρδw02·exp⁡sgnlilθexp⁡sgnl−imθ

(6) Emρ,φ=2m!π1w0ρ2w0mexp⁡−ρ2w02·exp⁡ilφ

and* I*_*m*_(*x*) is the* m*th-order modified Bessel function of the first kind.

The derivation of the above OAM spectrum is similar to that in [19], where an on-axis LG beam was expanded into a set of weighted off-axis LG beams and the OAM spectrum is symmetric with respect to the original mode* l*.

*Parameters That Affect the Imaging Plane When an Off-Axis Point Is Encoded/Decoded.* As indicated by (4)-(6), complex field of the imaging plane when an off-axis point is encoded/decoded can be affected with different total OAM mode *l* imposed, displacement of the off-axis point* δ*, angular position of the off-axis point *θ*, and beam waist of the modelled LG beams *w*_0_. Intensity of the imaging plane, however, is not affected by *θ*. All the parameters are the same as that in the experiment, except for the beam waist *w*_0_, which is related to the beam size incident on the plane *U*_*d*_1__(*ξ*, *η*) (Figure 6(a)). The smaller the beam size, the larger the *w*_0_.

*Measuring Intensity Distribution of the Central Component in an OAM Spectrum by Measuring Intensity of the Imaging Plane When a Thin Ring Is Encoded/Decoded.* Suppose a thin ring at *ρ* = *δ* is encoded/decoded with a total spiral mode index l, the resultant complex field at the imaging plane can be summed/integrated over all of the off-axis crosstalk functions generated by the off-axis points positioned at *ρ* = *δ*, with the angular position *θ* = 0 ~ 2*π*, yielding

(7) Uring,lρ,φ=∑m=−∞∞aEsgnlmρ,φ∫02πBsgnlmρ,φdθ=2aπ×Bsgnlmρ,φEsgnlmρ,φm=l

where *a* is amplitude of the ring. The central mode *m* = *l* in an OAM spectrum is also the main component when *δ* is sufficiently small [19]. Equation (7) indicates that intensity of the imaging plane when a ring is encoded/decoded is 4*a*^2^*π*^2^ times that of the central component (and the main one in our experiment) of an OAM spectrum.

*Data Processing.* In Figure 1(b), decomposition of an on-axis OAM beam into off-axis OAM spectra was achieved by decomposing the Gaussian profile into weighted helical profiles and decomposing the helical phase into weighted modified Bessel functions [25].

In Figures 2 and 4, the origin of a coordinate system was found by averaging central positions of horizontal and vertical cross-sections. In Figure 2(c1), peak radius was the radius at maximum intensity and 10% radius was found at 10% of maximum intensity.

In Figure 3(c1), sharpness of the edges was calculated by* dI*/*dx* at intersections with green dashed line.

## References

[1] Levoy M., Hanrahan P. Light field rendering.

[2] Yu H., Lee K., Park J., Park Y. (2017). Ultrahigh-definition dynamic 3D holographic display by active control of volume speckle fields. *Nature Photonics*.

[3] Blanche P.-A., Bablumian A., Voorakaranam R. (2010). Holographic three-dimensional telepresence using large-area photorefractive polymer. *Nature*.

[4] Jia J., Chen J., Yao J., Chu D. (2017). A scalable diffraction-based scanning 3D colour video display as demonstrated by using tiled gratings and a vertical diffuser. *Scientific Reports*.

[5] Hall D. R. Use of stereoscopic systems utilising chiral liquid crystals.

[6] Fattal D., Peng Z., Tran T. (2013). A multi-directional backlight for a wide-angle, glasses-free three-dimensional display. *Nature*.

[7] Chen W. T., Yang K., Wang C. (2013). High-efficiency broadband meta-hologram with polarization-controlled dual images. *Nano Letters*.

[8] Ramos-Diaz E., Ponomaryov V. (2011). 3D video visualization employing wavelet multilevel decomposition. *Applied Optics*.

[9] Morgan M. (1975). Stereoillusion based on visual persistence. *Nature*.

[10] Chien K.-W., Shieh H.-P. D. (2006). Time-multiplexed three-dimensional displays based on directional backlights with fast-switching liquid-crystal displays. *Applied Optics*.

[11] Peterka T., Kooima R. L., Sandin D. J., Johnson A., Leigh J., DeFanti T. A. (2008). Advances in the dynallax solid-state dynamic parallax barrier autostereoscopic visualization sisplay system. *IEEE Transactions on Visualization and Computer Graphics*.

[12] Zhao W., Wang Q., Wang A., Li D. (2010). Autostereoscopic display based on two-layer lenticular lenses. *Optics Expresss*.

[13] Gibson G., Courtial J., Padgett M. J. (2004). Free-space information transfer using light beams carrying orbital angular momentum. *Optics Express*.

[14] Lei T., Zhang M., Li Y. (2015). Massive individual orbital angular momentum channels for multiplexing enabled by Dammann gratings. *Light: Science & Applications*.

[15] Wang J., Yang J.-Y., Fazal I. M. (2012). Terabit free-space data transmission employing orbital angular momentum multiplexing. *Nature Photonics*.

[16] Yan Y., Xie G., Lavery M. P. J. (2015). High-capacity millimetre-wave communications with orbital angular momentum multiplexing. *Nature Communications*.

[17] Yao A. M., Padgett M. J. (2011). Orbital angular momentum: origins, behavior and applications. *Advances in Optics and Photonics*.

[18] Allen L., Beijersbergen M. W., Spreeuw R. J. C., Woerdman J. P. (1992). Orbital angular momentum of light and the transformation of Laguerre-Gaussian laser modes. *Physical Review A: Atomic, Molecular and Optical Physics*.

[19] Bazhenov V., Vasnetsov M. V., Soskin M. S. (1990). Laser-beams with screw dislocations in their wave-fronts. *JETP Letters*.

[20] Padgett M. J., Miatto F. M., Lavery M. P. J., Zeilinger A., Boyd R. W. (2015). Divergence of an orbital-angular-momentum-carrying beam upon propagation. *New Journal of Physics*.

[21] Krenn M., Handsteiner J., Fink M. (2014). Twisted light communication through turbulent air across Vienna. *New Journal of Physics*.

[22] Li X., Chu J., Smithwick Q., Chu D. (2016). Automultiscopic displays based on orbital angular momentum of light. *Journal of Optics (United Kingdom)*.

[23] Graydon O. (2016). 3D displays: Momentum multiplexing. *Nature Photonics*.

[24] Chu J., Li X., Smithwick Q., Chu D. (2016). Coding/decoding two-dimensional images with orbital angular momentum of light. *Optics Expresss*.

[25] Chu J., Chu D., Smithwitck Q. (2017). Off-axis points encoding/decoding with orbital angular momentum spectrum. *Scientific Reports*.

[26] Vasnetsov M. V., Pas'ko V. A., Soskin M. S. (2005). Analysis of orbital angular momentum of a misaligned optical beam. *New Journal of Physics*.

[27] Cvijetic N., Milione G., Ip E., Wang T. (2015). Detecting lateral motion using light’s orbital angular momentum. *Scientific Reports*.

[28] Fürhapter S., Jesacher A., Bernet S., Ritsch-Marte M. (2005). Spiral phase contrast imaging in microscopy. *Optics Express*.

[29] Situ G., Warber M., Pedrini G., Osten W. (2010). Phase contrast enhancement in microscopy using spiral phase filtering. *Optics Communications*.

[30] Oemrawsingh S. S. R., Van Houwelingen J. A. W., Eliel E. R. (2004). Production and characterization of spiral phase plates for optical wavelengths. *Applied Optics*.

[31] Uchida M., Tonomura A. (2010). Generation of electron beams carrying orbital angular momentum. *Nature*.

[32] Zhang Z., You Z., Chu D. (2014). Fundamentals of phase-only liquid crystal on silicon (LCOS) devices. *Light: Science & Applications*.

[33] Zhu L., Wang J. (2014). Arbitrary manipulation of spatial amplitude and phase using phase-only spatial light modulators. *Scientific Reports*.

[34] Goodman J. W. (2005). *Introduction to Fourier Optics*.

[35] Geng J. (2013). Three-dimensional display technologies. *Advances in Optics and Photonics*.

[36] Liao H. (2015). Super long viewing distance light homogeneous emitting three-dimensional display. *Scientific Reports*.

[37] Dodgson N. A. (2005). Autostereoscopic 3D displays. *The Computer Journal*.

[38] Son J.-Y., Javidi B. (2005). Three-dimensional imaging methods Based on multiview images. *Journal of Display Technology*.

[39] Takaki Y., Nago N. (2010). Multi-projection of lenticular displays to construct a 256-view super multi-view display. *Optics Express*.

[40] Drevinskas R., Kazansky P. G. (2017). High-performance geometric phase elements in silica glass. *APL Photonics*.

[41] Yao E., Franke-Arnold S., Courtial J., Barnett S., Padgett M. (2006). Fourier relationship between angular position and optical orbital angular momentum. *Optics Express*.

[42] Jha A. K., Jack B., Yao E. (2008). Fourier relationship between the angle and angular momentum of entangled photons. *Physical Review A: Atomic, Molecular and Optical Physics*.

[43] Karimi E., Schulz S. A., De Leon I., Qassim H., Upham J., Boyd R. W. (2014). Generating optical orbital angular momentum at visible wavelengths using a plasmonic metasurface. *Light: Science & Applications*.

